# Aspect-Object Alignment with Integer Linear Programming in Opinion Mining

**DOI:** 10.1371/journal.pone.0125084

**Published:** 2015-05-22

**Authors:** Yanyan Zhao, Bing Qin, Ting Liu, Wei Yang

**Affiliations:** 1 Department of Media Technology and Art, Harbin Institute of Technology, Harbin, China; 2 Department of Computer Science and Technology, Harbin Institute of Technology, Harbin, China; 3 Intelligent Computing and Search Lab, Tencent, Beijing, China; National Center for Biotechnology Information, UNITED STATES

## Abstract

Target extraction is an important task in opinion mining. In this task, a complete target consists of an aspect and its corresponding object. However, previous work has always simply regarded the aspect as the target itself and has ignored the important "object" element. Thus, these studies have addressed incomplete targets, which are of limited use for practical applications. This paper proposes a novel and important sentiment analysis task, termed aspect-object alignment, to solve the "object neglect" problem. The objective of this task is to obtain the correct corresponding object for each aspect. We design a two-step framework for this task. We first provide an aspect-object alignment classifier that incorporates three sets of features, namely, the basic, relational, and special target features. However, the objects that are assigned to aspects in a sentence often contradict each other and possess many complicated features that are difficult to incorporate into a classifier. To resolve these conflicts, we impose two types of constraints in the second step: intra-sentence constraints and inter-sentence constraints. These constraints are encoded as linear formulations, and Integer Linear Programming (ILP) is used as an inference procedure to obtain a final global decision that is consistent with the constraints. Experiments on a corpus in the camera domain demonstrate that the three feature sets used in the aspect-object alignment classifier are effective in improving its performance. Moreover, the classifier with ILP inference performs better than the classifier without it, thereby illustrating that the two types of constraints that we impose are beneficial.

## Introduction

The Web contains a considerable amount of user-generated content that describes the opinions of customers regarding products and services in the form of reviews, blogs, tweets, and so on. These reviews are valuable for assisting customers in making purchasing decisions and for guiding companies in pursuing their business activities. However, browsing the extensive collection of available reviews to search for useful information is a time-consuming and tedious task. Consequently, sentiment analysis and opinion mining have attracted significant attention in recent years because they provide an approach to the automatic analysis of user reviews and the extraction of the information that is most relevant to users [1].

Opinion mining and sentiment analysis entail a number of interesting and challenging tasks, such as sentiment classification [2–6], sentiment extraction [7–11] and sentiment summarization [12–14]. One fundamental task is target extraction [8, 9, 15, 16], with the intent of identifying the main topic that has been commented on in a review. Generally speaking, a target consists of an object and an aspect, where the object refers to the product brand and the aspect refers to the attribute or feature of a certain product. For example, in the review “I bought a [*Canon* 600*D*]^*o*^ yesterday, and its [*photos*]^*a*^ are amazing,” the complete target is ⟨*photos*, *Canon* 600*D*⟩, where “*Canon 600D*,” which is a digital camera brand, is the object, and “*photos*,” which is an attribute of a digital camera, is the aspect. However, previous work has always considered the aspect alone as the target [17–20], and thus, the considered target has not been complete. For instance, in the aforementioned review, the aspect “*photos*” in the second sentence would always have been directly tagged as the target. Clearly, this target is incomplete because it ignores a very important element, namely, the object, “*Canon 600D*,” to which the aspect “*photos*” belongs. We refer to this problem as “object neglect.” An incomplete target is of limited use for practical applications if the object is not recognized.

In this paper, we define the target as consisting of two parts, namely, the aspect and its corresponding object, such as ⟨*photos*, *Canon* 600*D*⟩. Correspondingly, the task of target extraction consists of two main subtasks:

- Aspect/object extraction, with the intent of extracting the aspects/objects in sentiment sentences. Please note that the objects that are relevant to a given domain are limited and can be manually collected. Therefore, few researchers have studied the task of automatic object extraction.
- ***Aspect-Object Alignment***, with the intent of determining the correct corresponding object for each aspect in the review.

At present, researchers predominantly focus on the first task and ignore the second task, even though the latter task is more important for practical applications. The target distribution statistics (400 reviews and 4,164 sentiment sentences from the camera and phone domains) in this study reveal that only 10% of aspects refer to objects in the same sentence, which is a very low rate of co-occurrence. This result illustrates that only a few aspects and their corresponding objects appear together in the same sentence, as in the case of the sentence “the [*appearance*]^*a*^ of [*GF*3]^*o*^ is very beautiful.” Rather, most aspects and their corresponding objects do not co-occur, as in the case of the sentence “the [*appearance*]^*a*^ is very beautiful.” In addition, the statistics also indicate that most reviews refer to more than one object. Therefore, we must explore the objects that are associated with aspects, most of which appear in other sentences, and choose the right one(s) from among several candidate objects. As seen from the above discussion, aspect-object alignment is both a new and necessary task. Thus, this task will be our primary focus in this paper. We hypothesize that all aspects and objects that appear in each sentiment sentence have been manually annotated.

We can regard the aspect-object alignment task as a simple binary classification, in which each decision is made in a pair-wise manner for each pair consisting of an aspect and an object, independently of the other pairs. Moreover, we propose three sets of features to incorporate into the aspect-object classifier, namely, the basic, relational, and special target features. However, there is one major shortcoming of this method, namely, that each decision is made independently of previous ones in a greedy manner. Clearly, the determination of the relation between an aspect and an object should be conditioned based on how well the approach functions as a whole. Because independence between decisions is an unwarranted assumption for this task, models that consider a more global context are likely to be more appropriate.

We address this issue by recasting the task of aspect-object alignment as an optimization problem, namely, an Integer Linear Programming (ILP) problem. ILP can be used to perform global inference based on the output of a classifier, which makes it a highly suitable method of addressing the aforementioned problem. First, we simply use ILP to search for a global assignment based on decisions obtained using the binary aspect-object classifier alone. The resulting assignment maximally agrees with the decisions of the classifier, in which each aspect corresponds to only one object. Second, we provide a joint formulation, in which constraints are added to ensure that the ultimate results are mutually consistent.

Two types of constraints are designed.

- **Intra-sentence constraints:** These describe the constraints between objects and aspects or between two aspects when the objects and aspects appear *in the same sentiment sentence*. For example, if a normal sentence (non-comparative sentence) contains two aspects but no object, then the two aspects share the same object.
- **Inter-sentence constraints:** These describe the constraints between objects and aspects or between two aspects when the objects and aspects appear *in different sentiment sentences*. This type of constraint must always rely on the concept of sentiment consistency, as inspired by the work of Ding et al. [21].

We evaluate our proposed framework on a review corpus in the digital camera domain. The experimental results reveal that the imposition of the two types of constraints described above allows us to achieve performances that are considerably higher than that of the base classifier. The joint model in particular achieves accuracy improvements of more than 5% over the cascading rule-based baseline and nearly 2% over the aspect-object alignment classifier.

The main contributions of this work can be summarized as follows.

- We propose a novel and important sentiment analysis task, termed aspect-object alignment, to solve the “object neglect” problem.
- We propose a two-step framework, which includes an aspect-object alignment classifier and ILP inference, to perform the aspect-object alignment task.
- Two types of constraints are introduced in the ILP procedure and can assist in generating optimal results.

The remainder of this paper is organized as follows. Section 2 presents an introduction to the related work. Section 3 describes the two-step framework, including the basic aspect-object alignment classifier and the ILP inference procedure with intra-sentence and inter-sentence constraints. Sections 4 and 5 present the experiments and results. Finally, we conclude the paper in Section 6.

## Related Work

### Target Extraction

Target extraction is an important task in sentiment analysis and has recently attracted considerable attention. Many efficient approaches have been developed for this task, and these approaches can be divided into three types of methods, namely, rule-based [15, 17, 18, 22], supervised [23–25], and topic-model-based [9, 26–28] methods. However, most researchers treat the aspects alone as the complete targets; thus, these algorithms are all designed specifically for aspect extraction. The “object” component of the target is ignored, and few researchers are studying the aspect-object alignment task.

The aspect-object alignment task is similar to the entity assignment task [21], which involves the assignment of objects to each sentence in a review. Many rules have been proposed for the simple execution of this task, and these rules primarily focus on processing comparative sentences based on the concept of sentiment consistency. The major problem with this approach is the sequential application of the rules, which sometimes leads to conflicts. Therefore, this method cannot effectively achieve an optimal result. In fact, this task is somewhat different from that addressed in our proposed approach, of which the objective is to find an object for each aspect. However, we can use a simple modification of this method as a baseline for comparison with our approach.

Several researchers have studied ontology construction from product reviews, one of the numerous subtasks of which is to recognize the relationships between aspects and objects. Wei et al. [14] proposed the concept of a “Sentiment Ontology Tree;” they constructed this tree manually and then proposed an HL-SOT approach to compute the associated sentiments for each attribute. Similarly, Yu et al. [23] generated a hierarchical organization of consumer reviews related to various product aspects and aggregated consumer opinions on these aspects. This product ontology construction task also differs from ours. This task comprises numerous subtasks, such as aspect extraction, sentiment computing, and hierarchical learning. Researchers have proposed several frameworks for this purpose but have not studied the algorithms for specific individual subtasks, particularly the aspect-object alignment task.

The aspect-object alignment task is also similar, to a certain extent, to the task of coreference resolution [29, 30], which has been extensively studied. In this task, an aspect can be treated as an anaphor, and an object can be treated as an entity. Recently, several researchers have studied coreference resolution in the context of review texts [31]. The most significant approach to this task is based on supervised learning, in which a pair-wise function is used to predict a coreferent pair of noun phrases. These methods serve as an inspiration for us to implement the learning of several useful features, such as distance features, for the aspect-object alignment task.

### ILP

An increasing interest in global inference methods for natural language processing has recently been observed. ILP-based models have been developed for many tasks, ranging from semantic role labeling [32] to multi-document summarization [33], and opinion mining [34]. Researchers formulate these tasks using the ILP approach, which has proven to be very effective in solving problems with complex constraints. These tasks inspire our formulation of the aspect-object alignment task, in which an ILP approach is used to attempt to optimize the relationships between aspects and objects by simultaneously considering intra-sentence and inter-sentence constraints.

The ILP-based approach possesses several properties that make it highly suitable for addressing the aspect-object alignment task. First, ILP can be used to perform global inference based on a classifier’s output. Second, it is more efficient than certain global inference algorithms, such as conditional random fields, especially when long-distance features are considered [35]. Finally, it is straightforward to create categorical global constraints using ILP, which can be imposed in a declarative manner by using equalities in the assignment to indicator variables.

## Method

This section introduces the proposed overall framework. The framework consists of two main steps: learning an aspect-object alignment classifier and applying ILP inference. Generally speaking, an aspect-object alignment classifier is learned to estimate the probability for each pair consisting of an aspect and an object. We apply an ILP inference procedure to achieve an optimal global result considering certain specific constraints.

### Aspect-Object Alignment Classifier

We can generate the Cartesian product of all aspects and objects contained in each review in the form of pair-wise vectors ⟨*a*, *o*⟩. The aspect-object alignment task can be formulated as the classification of each ⟨*a*, *o*⟩ as true or false. The classification approach addresses aspect-object alignment in two steps as follows: (1) estimating the probability, *P*_*align*_(*ALIGN*∣⟨*a*, *o*⟩), of a certain alignment outcome given an aspect-object pair ⟨*a*, *o*⟩ and (2) applying a selection algorithm that will single out a unique candidate from among the subset of candidates *o* for which the probability *P*_*align*_(*ALIGN*∣⟨*a*, *o*⟩) meets or exceeds a particular value.

We use a maximum entropy model for the aspect-object alignment classifier. Such models are well-suited for this task because they are able to handle many different, potentially overlapping learning features without requiring any assumptions regarding independence. Maximum entropy has been used as a classifier in many studies that have achieved notably high performances, for tasks including coreference resolution, word sense disambiguation, and text classification, among others. The model can be defined in a standard form as follows, where *Z*(⟨*a*, *o*⟩) is a normalization factor for both outcomes (*ALIGN* and ¬*ALIGN*).

$$\begin{array}{c} P_{Align(ALIGN|\langle a,o\rangle )}=\frac{exp(\sum_{k=1}^{n}\lambda_{k}f_{k}\left(\left\langle a,o\right\rangle ,ALIGN\right))}{Z(\langle a,o\rangle )} \end{array}$$ (1)

**Fig 1:**
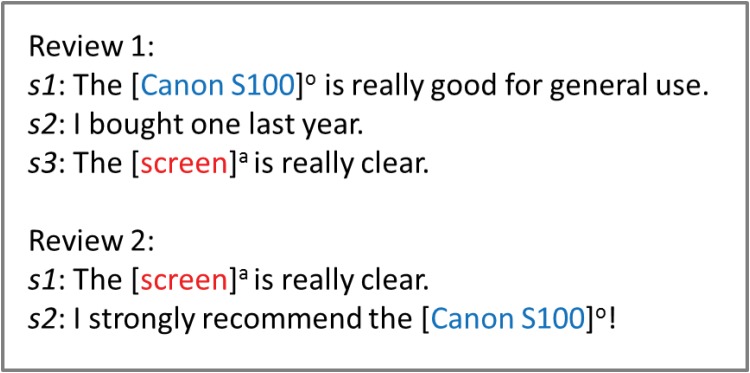
Example of the three types of sentences.

We design the features for classifying each ⟨*a*, *o*⟩ extracted from three types of relative sentences, namely, *the present sentence*, *the previous sentence* and *the nearest sentence*. The definitions of these three sentence types are provided below. We also present some examples in Fig 1 to better illustrate these sentence types.

- Present sentence: this type of sentence contains an aspect *a*, such as *s*_3_ of review 1 and *s*_1_ of review 2 in Fig 1.
- Previous sentence: this type of sentence must satisfy two conditions: (1) it contains an object, and (2) it is the nearest preceding sentence of interest to the present sentence. For example, *s*_1_ of review 1 is the previous sentence of the present sentence *s*_3_. We explore the features generated from this type of previous sentence because in certain cases, the corresponding object for the *a* that appears in the present sentence can be acquired from the previous sentence. For example, the object “*Canon S100*” in the previous sentence *s*_1_ of review 1 is the object that corresponds to the aspect “*screen*” in the present sentence *s*_3_.
- Nearest sentence: this type of sentences must also satisfy two conditions: (1) it contains an object, and (2) it is the nearest sentence to the present sentence. Note that the nearest sentence is different from the previous sentence because sometimes it appears after the present sentence. It should be noted that we assign a high priority to the sentence before the present sentence, when two sentences appear before and after the present sentence respectively and have the same distance. We consider the features generated from the nearest sentence because in certain cases, the object that corresponds to the *a* in the present sentence can be acquired from the nearest sentence. For example, in review 2 of Fig 1, the nearest sentence with respect to the present sentence is *s*_2_, and the object that corresponds to the aspect “*screen*” in *s*_1_ is “*Canon S100*” in *s*_2_.

Based on the three types of sentences, we propose three categories of features, namely, basic features, relational features and special target features, as follows:

**Basic Features:**

Here, we formulate the several basic features that can be obtained from the present, previous and nearest sentences.

- **Sentence-type feature**: Sentiment sentences can be divided into three types based on the objects or aspects they contain. The first type of sentence contains one or more objects but no aspect, for example, “The [*CanonS*100]^*o*^ is really good for general use.” The second type of sentence contains both objects and aspects, for example, “The [*screen*]^*a*^ of the [*CanonS*100]^*o*^ is really clear.” The third type of sentence contains one or more aspects but no object. For example, “The [*screen*]^*a*^ is really clear.” The possible encoded values of these types of sentences are 01, 02 and 03, respectively. This feature is used to describe the present, previous, nearest sentences. We incorporate this feature because the methods for aspect-object alignment vary for different types of sentences.
- **Comparative sentence feature**: Sentiment sentences can be divided into normal and comparative sentences. If the present/previous/nearest sentence is a comparative sentence, then the value is true; otherwise, the value is false. We incorporate this feature because the method for aspect-object alignment for normal sentences is different from that for comparative sentences. It should be noted that some previous work has focused on the identification of comparative sentences [36]. Our focus in this paper is not on identifying such sentences; thus, we apply several heuristic rules based on the presence of several comparative keywords to identify comparative sentences, e.g., 比, 比较, 相比(“than” in English), etc.
- **Object feature**: This feature refers to the object that appears in the present/previous/nearest sentence.

**Relational Features:**

We also formulate two types of relational features. One type includes distance features, the inclusion of which was inspired by the coreference resolution task [31]. The other type includes object consistency features, the inclusion of which was inspired by Ding et al. [21].

- **Distance between present and previous sentence**: The possible values are 0, 1, 2, 3, etc., where the value represents the number of sentences between the present sentence and its previous sentence.
- **Distance between present and nearest sentence**: The possible values are 0, 1, 2, 3, etc., where the value represents the number of sentences between the present sentence and its nearest sentence.
- **Consistency between the object in the previous sentence and the candidate object in ⟨*a*, *o*⟩**: If the object in the previous sentence is the same as the one in ⟨*a*, *o*⟩, then the value is true; otherwise, the value is false.
- **Consistency between the object in the nearest sentence and the candidate object in ⟨*a*, *o*⟩**: If the object in the nearest sentence is the same as the one in ⟨*a*, *o*⟩, then the value is true; otherwise, the value is false.

**Special Target Features:**

- **First appearing object in the review**: This feature refers to the first object to appear in the review.
- **Most frequent object in the review**: This feature refers to the object that appears most frequently in the review.

Using the above features, we can construct an aspect-object alignment classifier to evaluate each ⟨*a*, *o*⟩ candidate.

### ILP Inference

In an ideal setting that has a perfect aspect-object alignment classifier, each aspect can be associated with the correct object based on the classifier’s prediction. In reality, labels (objects) that are assigned to aspects in a sentence often contradict each other and violate the constraints that arise from the available structural and linguistic information. For example, each aspect corresponds to only one object. These complicated features are difficult to incorporate into a classifier. Therefore, we encode the relevant constraints as linear formulations to resolve the conflicts. We also apply an ILP-based inference procedure to produce a final decision that is consistent with the constraints. The confidence scores for each ⟨*a*, *o*⟩ pair that are produced by the aspect-object alignment classifier are used as the input to produce the best global alignments that satisfy the constraints.

We formally define the aspect set as *A* = {*a*_1_, *a*_2_, ……, *a*_*n*_} and the object set as *O* = {*o*_1_, *o*_2_, ……, *o*_*m*_} for each review. We assume that the resulting object set for the aspects in *A* is *S* = {*s*_1_, *s*_2_, ……, *s*_*n*_}, where *s*_*i*_ ∈ *O* and represents the resulting object corresponding to *a*_*i*_. Thus, this task can be formulated such that the aspect-object assignment classifier attempts to assign an object from *O* to each *a*_*i*_ in set *A*. If we assume that the classifier returns a probability value *p*(*a*_*i*_, *s*_*i*_), which corresponds to the likelihood of assigning label *s*_*i*_ to aspect *a*_*i*_, then the inference task for a given review can be formulated as the maximization of the overall score of the aspects as follows, where $\hat{S}$ is the optimal result among all potential vectors.

$$\begin{array}{c} \hat{S}=argmax\sum_{i=1}^{n}p\left(a_{i},s_{i}\right) \end{array}$$ (2)

In this equation, the probability value *p*(*a*_*i*_, *s*_*i*_) can be obtained from the aforementioned aspect-object alignment classifier introduced in Section 3.1. If *s*_*i*_ is the *j* ^*th*^ object in the object set *O*, then *p*(*a*_*i*_, *s*_*i*_) can be denoted by *p*_*ij*_, which represents the probability value for the pair consisting of the *i* ^*th*^ aspect in *A* and the *j* ^*th*^ object in *O*.

We can introduce a set of binary indicator variables *z*_*ij*_ ∈ {0, 1} for each *p*_*ij*_ to reformulate the original $\hat{S}$ function into a linear function to assist in acquiring the optimal $\hat{S}$. If the resulting object corresponding to *a*_*i*_ is actually *o*_*j*_, then the value of the corresponding variable is *z*_*ij*_ = 1; otherwise, *z*_*ij*_ = 0. Thus, the task of finding an optimal $\hat{S}$ can be converted into the task of finding an optimal vector *Z* that maximizes the objective function. Here, *Z* is the set of *z*_*ij*_, where *i* ∈ {1, 2, ……, *n*} and *j* ∈ {1, 2, ……, *m*}. Eq (2) can be written as an ILP objective function as follows:

$$\begin{array}{c} \hat{Z}=argmax\sum_{i=1}^{n}\sum_{j=1}^{m}p_{ij}z_{ij} \end{array}$$ (3)

Subject to

$$\begin{array}{c} \sum_{j=1}^{m}z_{ij}=1,\;\;\;\;\;\;\;\;\;\forall z_{ij}\in Z \end{array}$$ (4)

Note that although this constraint originates from the variable transformation, it has a practical interpretation, namely, that each aspect can take only one object. This constraint is referred to as **constraint 1**.

Next, we impose several constraints on Eq (3) to acquire the optimum solution. The designed constraints can be divided into two categories. The first category focuses on describing the constraints between objects and aspects or between two aspects when the objects and aspects appear in the *same* sentiment sentence. We refer to this category, which comprises four constraints in total and includes constraint 1, as **intra-sentence constraints**. The second category focuses on describing the constraints between objects and aspects or between two aspects when the objects and aspects appear in *different* sentences. We refer to this category, which comprises two constraints, as **inter-sentence constraints**. These constraints will be introduced individually in the next section.

#### Intra-sentence constraints

Constraints 2 through 4 constitute the remaining intra-sentence constraints.

**Constraint 2**: If aspects *a*_*p*_ and *a*_*q*_ appear in the same sentence *s* and no object appears in the sentence, then *a*_*p*_ has the same object as that of *a*_*q*_. For example, in the sentence “It has a good [*resolution*]^*a*^ and a good [*LCD* *screen*]^*a*^,” the aspects “*resolution*” and “*LCD screen*” satisfy this constraint; thus, they have the same object. This constraint is represented by the following equation:

$$\begin{array}{c} \forall j\in \left\{1,......,m\right\}:\;\;\;\;z_{pj}=z_{qj}, \end{array}$$ (5)

where two aspects *a*_*p*_ and *a*_*q*_, but no object appear in *s*.

**Constraint 3**: This constraint is relevant only when the given sentence *s* satisfies three conditions: (1) *s* is a comparative sentence; (2) *s* contains two objects, *o*_*k*_ and *o*_*t*_, which appear on opposite sides of the comparative word; and (3) only one aspect, *a*_*p*_, appears in the sentence. Then, the object that corresponds to *a*_*p*_ is one of the two objects that appear in the given sentence. For example, in the sentence “the [*shutter* *sound*]^*a*^ of the [*Canon* 5*D*3]^*o*^ is better than the [5 *D* 2]^*o*^’s,” the aspect “*shutter sound*” must belong to one of the objects that appear in this sentence. In this case, the object in question is the “*Canon 5D3*.” This constraint can be described using the following formulation:

$$\begin{array}{c} z_{pk}+z_{pt}=1, \end{array}$$ (6)

where only one aspect, *a*_*p*_, and two objects, *o*_*k*_ and *o*_*t*_, appear in the comparative sentence *s* and *o*_*k*_ and *o*_*t*_ appear on opposite sides of the comparative word.

**Constraint 4**: This constraint is relevant only when the given sentence *s* satisfies two conditions: (1) *s* contains only one aspect, *a*_*p*_, and one object, *o*_*k*_; and (2) the sentence is a normal sentence. Then, the object that corresponds to *a*_*p*_ is the object *o*_*k*_. For example, in the sentence “the [*shutter* *sound*]^*a*^ of the [*Canon* 5*D*3]^*o*^ is good,” “*Canon 5D3*” is the correct object associated with the aspect “*shutter sound*.” This constraint can be described using the following formulation:

$$\begin{array}{c} z_{pk}=1, \end{array}$$ (7)

where only one aspect *a*_*p*_ and one object *o*_*k*_ appear in the normal sentence *s*.

#### Inter-sentence constraints

Many previous studies have demonstrated that adjacent sentences in a review have particular sentiment relationships [21, 37]. Thus, the sentiment orientations for two adjacent sentences are always either the same or entirely opposite (as indicated by the usage of transitional words, such as “but”); this phenomenon is known as “sentiment consistency.” Zhao et al. [37] used this rule to improve performance in the sentence sentiment classification task. Ding et al. [21] used this concept as an important feature to perform the object and attribute coreference task. This concept is also very useful in the aspect-object alignment task. We formulate two constraints based on this concept as follows.

**Constraint 5**: This constraint is relevant only when the given sentence *s*_*g*_ satisfies three conditions: (1) *s*_*g*_ contains only an aspect, *a*_*p*_, and no object; (2) the previous sentence, *s*_*p*_, is a normal sentence that contains an aspect, *a*′, and an object, *o*_*k*_; and (3) the sentiment orientation of *s*_*g*_ is the same as that of *s*_*p*_. Then, the object that corresponds to *a*_*p*_ is the object *o*_*k*_ that appears in the previous sentence *s*_*p*_. For example, in the sentences “The best feature of the [*Canon* *S*110]^*o*^ is its [*size*]^*a*^. The [*picture* *quality*]^*a*^ is good, too,” the second sentence satisfies the necessary conditions for this constraint to apply and the object that corresponds to the aspect “*picture quality*” is the object “*Canon S110*” that appears in the previous sentence. This constraint can be described using the following formulation:

$$\begin{array}{c} z_{pk}=1, \end{array}$$ (8)

where only one aspect, *a*_*p*_, but no object appears in the given sentence *s*_*g*_; only one aspect, *a*′, and one object, *o*_*k*_, appear in the normal previous sentence *s*_*p*_; and *polarity*(*s*_*g*_) = *polarity*(*s*_*p*_).

**Constraint 6**: This constraint is relevant only when the given sentence satisfies two conditions: (1) the given sentence *s*_*g*_ contains an aspect, *a*_*p*_, but no object; and (2) the previous sentence, *s*_*p*_, is a comparative sentence and contains two objects, *o*_*k*_ and *o*_*t*_, which appear on opposite sides of the comparative word such that *o*_*k*_ appears before *o*_*t*_. If the given sentence possesses the same sentiment orientation as does the previous sentence, then the object that corresponds to *a*_*p*_ is the object *o*_*k*_ that appears in the previous sentence. However, if the given sentence possesses a different sentiment orientation from that of the previous sentence, then the object that corresponds to *a*_*p*_ is *o*_*t*_.

For example, consider the review “the [*shutter* *sound*]^*a*^ of the [*Canon* 5*D*3]^*o*^ is better than the [5*D*2]^*o*^’s. The [*picture* *quality*]^*a*^ is good, too.” Here, the object that corresponds to “*picture quality*” in the second sentence is “*Canon 5D3*” in the previous sentence, not “*5D2*.” This constraint can be described using the following formulation:

$$\begin{array}{c} z_{pk}=1,\;\;\;\;\;\;\;\;\;\;\;\;\;\;if\;polarity\left(s_{g}\right)=polarity\left(s_{p}\right) \end{array}$$ (9)

$$\begin{array}{c} z_{pt}=1,\;\;\;\;\;\;\;\;\;\;\;\;\;\;if\;polarity\left(s_{g}\right)=-polarity\left(s_{p}\right), \end{array}$$ (10)

where only one aspect, *a*_*p*_, but no object appears in the given sentence *s*_*g*_ and two objects, *o*_*k*_ and *o*_*t*_, appear in the previous comparative sentence *s*_*p*_ and on opposite sides of the comparative word.

The intra-sentence and inter-sentence relationships discussed in the preceding sections can be encoded as constraints in the ILP inference process. With the problem formulated in this manner, we can use the aspect-object alignment classifier and also jointly apply an ILP model and many useful constraints to generate the optimal results.

## Experimental Setup and Baselines

### Corpus

We manually collected on-line customer reviews in the digital camera domain to use in a case study for the aspect-object alignment task. The corpus was obtained from two well-known Chinese forum sites, namely, http://ww.xitek.com/ and http://www.fengniao.com/. The statistics of the corpus are summarized in Table 1.

**Table 1:** Statistics of the corpus.

No.	Types	Digital camera
1	# of reviews	200
2	# of sentences	8,042
3	# of aspects	2,017
4	Average # of objects per review	2.82
5	# of pairwise ⟨*a*, *o*⟩	9,161

The raw corpus contains 200 documents. Each document includes the main body and title of the associated post. We asked two experts to annotate the aspects and objects in each sentence and to simultaneously annotate the polarity of the sentence. A total of 8,042 sentences were annotated. The calculated agreement according to Cohen’s *κ* score was found to be *κ* > 0.75, which is satisfactory. We applied a third independent annotation, if inconsistency was detected, to extend our dataset.

We summarize some statistics of the corpus in Table 1, which reports the presence of 2,017 aspects in the corpus and an average of 2.82 objects per review. According to the Cartesian product of all aspects and objects, all reviews in the corpus contain a total of 9,161 aspect-object pairs, which also serve as the input to the binary aspect-object alignment classifier.

**Ethics Statement** Please note that although we asked two experts to manually annotate the corpus, this type of research does not involve any privacy concerns related to the human participants. Moreover, these two experts are also co-authors of our paper, Bing Qin and Ting Liu. Therefore, the two experts consented to participate in this study. Besides, our collection of reviews was in compliance with the terms of use for the xitek and Fengniao websites. All data was publicly available.

### Baselines

**Fig 2:**
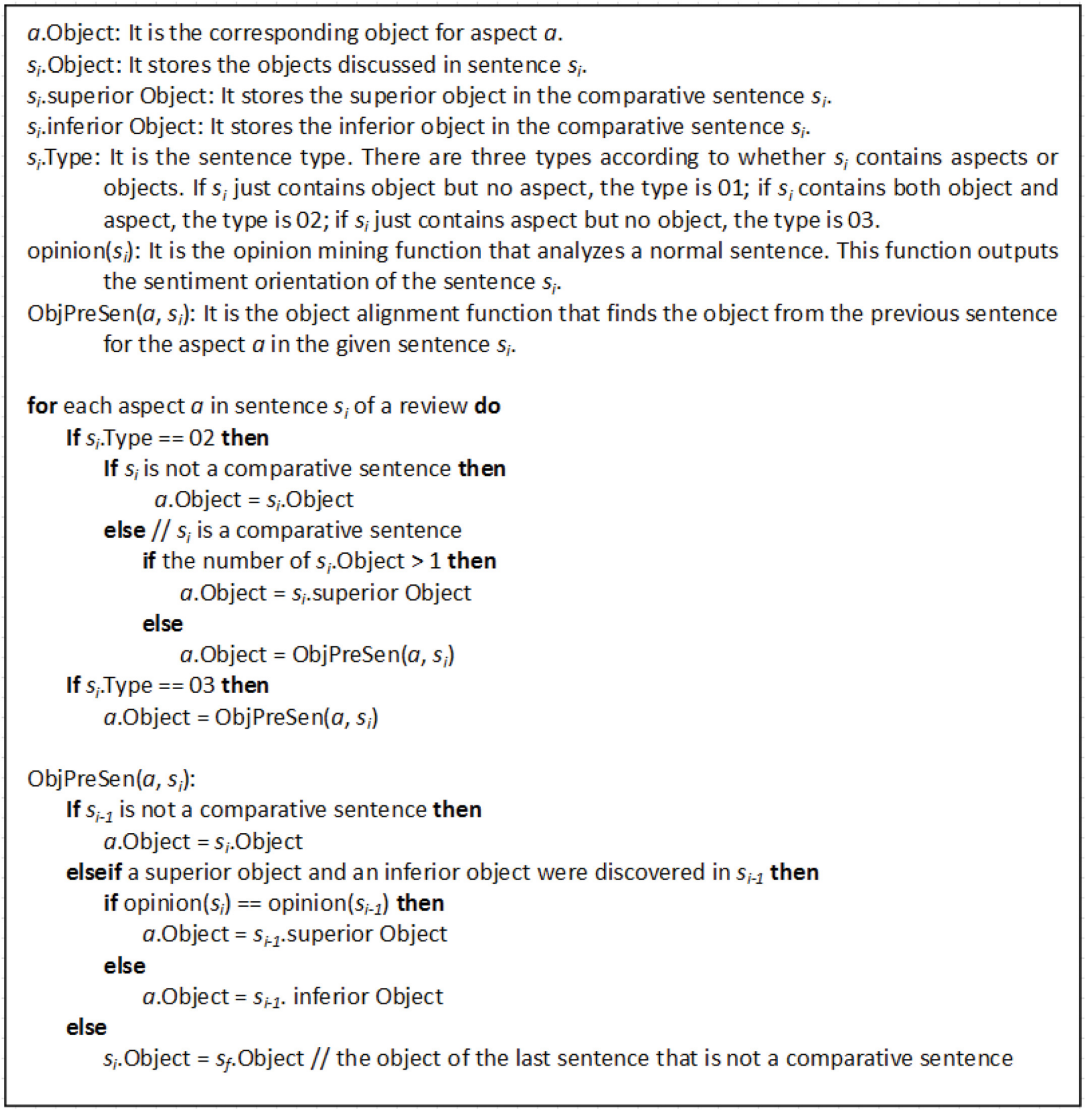
Algorithm for the cascading rule-based approach (Baseline1).

We compare our system with two baselines. **Baseline 1** is a cascading rule-based approach, which is similar to the method of Ding et al. [21]. We describe the cascading rule-based approach in detail in Fig 2. This approach combines several useful rules. For example, one simple rule is that if a non-comparative sentiment sentence contains an object and an aspect, then the object and aspect can be aligned. This method also uses the concept of sentiment consistency. The following example is provided for illustration: “The [*screen*]^*a*^ of the [*CanonS*110]^*o*^ is clearer than the [*S*100]^*o*^’s. Its [*pictures*]^*a*^ are really good, too.” We observe that the two objects “*Canon S110*” and “*S100*” are located before the aspect “*pictures*.” The comment regarding the “*Canon S110*” is positive and that regarding the “*S100*” is negative, whereas the comment on “*pictures*” is positive. The object “*Canon S110*” that exhibits the sentiment that is consistent with that of the aspect “*pictures*” can be considered to be the object that is associated with “*pictures*.” The primary shortcoming of this cascading rule-based approach is that several rules can conflict with one another during processing. **Baseline 2** is the aspect-object alignment classifier without ILP inference.

### Training and Evaluation

We experiment with the ME algorithm and tune the related parameters of the aspect-object alignment classifier by applying 10-fold cross-validation to the corpus described in the previous section. The ME tool used in this paper is available from Zhang [38].

We evaluate this task in two ways. To evaluate the usefulness of the features in the aspect-object alignment classifier, the classic Precision (*P*), Recall (*R*) and F-score (*F1*) values, which are defined as follows, are used to evaluate the performance in the binary classification of ⟨*a*, *o*⟩ pairs. There are two types of ⟨*a*, *o*⟩ pairs, namely, positive instances and negative instances. The meaning of “positive” in “**positive instances**” is different from the meaning of “positive” in sentiment analysis. Here, positive instances refer to the standard (annotated) ⟨*a*, *o*⟩ pairs in the corpus. Table 1 shows that the number of positive instances is equal to the number of aspects, which is 2,017. Similarly, the meaning of “negative” in “**negative instances**” is different from the meaning of “negative” in sentiment analysis. Here, negative instances refer to the incorrect (unannotated) ⟨*a*, *o*⟩ pairs in the corpus. Table 1 shows that there are 9,161 candidate aspect-object pairs in total, according to the Cartesian product of all aspects and objects, which includes both positive and negative instances. Therefore, the number of negative instances is 7,144.

$$\begin{array}{c} P=\frac{\#\;of\;correctly\;classified\;positive\;(negative)\;instances}{\#\;of\;classified\;positive\;(negative)\;instances} \end{array}$$ (11)

$$\begin{array}{c} R=\frac{\#\;of\;correctly\;classified\;positive\;(negative)\;instances}{\#\;of\;positive\;(negative)\;instances} \end{array}$$ (12)

$$\begin{array}{c} F1=\frac{2*P*R}{P+R} \end{array}$$ (13)

For the final results, because our objective in the aspect-object alignment task is to find the correct object for each aspect, the value *Accuracy* defined as follows is suitable for evaluating the success of this task.

$$\begin{array}{c} Accuracy=\frac{\#\;of\;aspects\;for\;which\;the\;correct\;objects\;can\;be\;found}{total\;\#\;of\;aspects\;in\;the\;corpus} \end{array}$$ (14)

## Results and Discussion

In this section, we first present the performances of our proposed ILP inference method and the two baselines that are described in Section 4.2. We also discuss the feature sets used in the aspect-object alignment classifier. Finally, we present and discuss the results obtained using the two types of constraints applied in the ILP inference procedure.

### Results of our method and the two baselines

Table 2 summarizes the performances of our ILP inference method and the two baselines, the cascading rule-based approach and the aspect-object alignment classifier.

**Table 2:** Comparison of the results of our method and two baselines.

Method	*Accuracy* (%)
Baseline 1: cascading rule-based	78.04
Baseline 2: aspect-object alignment classifier	81.80
Our ILP inference method	**83.69**

Table 2 shows that the performance of the cascading rule-base approach is far from ideal, which can be attributed to its nature as a rule-based method, in which the rules are applied sequentially with conflicts among them. Baseline 2, which is the aspect-object alignment classifier, achieves an accuracy of 81.80%, thus significantly (*χ* test with *p* < 0.0001) outperforming the cascading rule-based approach. The features incorporated in this method, which can provide a global description of the task, yield a considerable difference.

Furthermore, our method, which combines the aspect-object alignment classifier with ILP inference processing, performs best and significantly (*χ* test with *p* < 0.03) outperforms both baselines. It is clearly evident that our method with ILP inference performs better than does the method without it (namely, Baseline 2). This finding demonstrates that the application of ILP inference is beneficial and that the two types of constraints proposed in this paper are effective.

### Effects of the three sets of features in the aspect-object alignment classifier

Table 3 presents the experimental results of constructing the aspect-object alignment classifier using different feature-set combinations. The construction of this classifier is the first step of our proposed method. We can observe the following:

- The first row presents the results for the classifier constructed without the basic features and shows that the performances decrease by more than 16% with respect to the *F*-score for positive instances and 3% with respect to the *F*-score for negative instances compared with the classifier that includes all features. This finding illustrates that the inclusion of the basic features is effective because these features, which include the sentence type, comparative information, and object information of the given, previous and nearest sentences, are the basic evidence on which the entire classification task is based, as proven in the work of Ding et al.
- The second row presents the results for the classifier constructed without the relational features and shows that the performances decrease by more than 16% with respect to the *F*-score for positive instances and 3% with respect to the *F*-score for negative instances compared with the classifier that includes all features. The relational features primarily describe certain relationships among the given, previous, and nearest sentences, such as the distance and consistency relationships. The use of this feature set was inspired by the coreference resolution task, and these results demonstrate that the inclusion of this feature set is also beneficial.
- The third row presents the results for the classifier constructed without the special target features and shows that the performances decrease by less than 1% with respect to the *F*-score for both positive and negative instances compared with the classifier that includes all features. This finding suggests that the effect of including the special target features, such as the first appearing target or the most frequent object, is not very significant.
- The aspect-object alignment classifier constructed using all features (row 4) performs the best.

**Table 3:** Results of using different feature-set combinations in the aspect-object alignment classifier.

Feature set	Positive instances^1^ (%)			Negative instances^2^ (%)		
	P	R	F	P	R	F
No Basic features.	69.24	60.04	64.15	89.17	92.52	90.80
No Relational features.	71.03	58.40	63.88	88.84	93.19	90.95
No Special target features.	81.47	77.92	79.50	93.82	94.97	94.38
All features	82.88	77.89	**80.20**	93.86	95.44	**94.63**

^1^ “Positive instances” is defined in Section 4.3.

^2^ Similarly, “negative instances” is also defined in Section 4.3.

### Aspect-object alignment with ILP inference

ILP is used to perform global inference based on the classifier’s output to resolve conflicts between rules. Six constraints are applied in this paper, of which Constraints 1 through 4 reflect the constraints that arise from information contained within a sentiment sentence, whereas Constraints 5 and 6 reflect the constraints that apply between different sentiment sentences. Table 4 summarizes the performances of the system under different constraints.

**Table 4:** Results of aspect-object alignment using different ILP constraints.

Constraints	ILP constraints	*Accuracy* (%)
Intra-sentence constraints	ILP-c1	81.80
	ILP-c2	81.85
	ILP-c3	81.90
	ILP-c4	82.65
Inter-sentence constraints	ILP-c5	82.45
	ILP-c6	82.05
All constraints	ILP-c1-6	**83.69**

Table 4 reveals the following:

- Constraint 1 represents the fact that one object can be assigned to each aspect; thus, the result obtained using this constraint is equivalent to that of the basic aspect-object alignment classifier.
- Constraints 2 through 4 are the intra-sentence constraints and enforce the alignment of aspect-object pairs based on the structural information (such as the relationships between aspects and objects) that is conveyed within a single sentence. The improvement achieved using Constraint 4 is larger than that achieved using any other constraint, thus illustrating the effectiveness of this constraint. Constraints 2 and 3 produce only slight increases in accuracy compared with the basic classifier without ILP inference because for most instances that satisfy these two constraints, the correct results can also be obtained using the basic classifier.
- Constraints 5 and 6 are the inter-sentence constraints and predominantly rely on the concept of sentiment consistency. Table 4 shows that the improvements achieved using Constraint 5 and Constraint 6 are both significant. These two constraints primarily focus on sentences that contain aspects but no objects. The statistics show that the proportion of this type of sentence in the corpus is approximately 90%, which is very high. The effectiveness of these two constraints demonstrates that for sentences of this type, we can seek the corresponding objects in the neighboring sentiment sentences. Moreover, this finding further demonstrates that applying the concept of sentiment consistency can improve the performance achieved in this task.
- We combine Constraints 1 through 6 for the final inference, thereby obtaining the overall best accuracy of 83.69%. The application of all constraints combined thus further improves the performance in the aspect-object alignment task by approximately 2%, significantly (*χ* test with *p* < 0.03) outperforming the aspect-object alignment classifier without ILP inference.

## Conclusion and Future Work

In this paper, we propose a novel and important sentiment analysis task, ***aspect-object alignment***, with the intent of resolving the “object neglect” problem in target extraction. This task is extremely important in that failure to resolve it will render the opinions discovered from user-generated content of limited use in practical applications.

We propose a two-step framework to execute this task. We first develop an aspect-object alignment classifier that incorporates three sets of features, namely, basic, relational and special target features. Experiments on a corpus in the camera domain reveal that this classifier significantly outperforms the cascading rule-based method and further demonstrate that the features included in the classifier are effective in improving its performance. We also propose that the task of aspect-object alignment be recast as an optimization problem and use ILP inference to obtain the global results to resolve the difficulties that arise as a result of the assumption of the independence of the decisions made using the classifier. In this inference procedure, we impose two types of constraints, namely, intra-sentence constraints and inter-sentence constraints, to acquire the optimum solution. The experimental results demonstrate that the aspect-object alignment classifier with the ILP inference performs better than does the classifier without it and also illustrate that the six constraints proposed in this paper are very useful in achieving this superior performance.

The work presented in this paper can be extended in many ways. One possible approach would be to enhance the algorithm at each step by seeking more useful features for the classifier or more useful constraints for the ILP inference procedure to improve the performance of the task. Another possible development would be to use ILP to further merge the current system with an aspect/object recognition system to create a more generally practical system. Moreover, the aspect-object relations in the aspect-object alignment task can be regarded as asymmetric relations between entities. Thus, a more general alternative would be to apply the framework proposed in this paper to solve other asymmetric-relation extraction problems that are subject to global constraints.
